# System Principles Governing the Organization, Architecture, Dynamics, and Evolution of Gene Regulatory Networks

**DOI:** 10.3389/fbioe.2022.888732

**Published:** 2022-05-12

**Authors:** Julio A. Freyre-González, Juan M. Escorcia-Rodríguez, Luis F. Gutiérrez-Mondragón, Jerónimo Martí-Vértiz, Camila N. Torres-Franco, Andrea Zorro-Aranda

**Affiliations:** ^1^ Regulatory Systems Biology Research Group, Program of Systems Biology, Center for Genomic Sciences, Universidad Nacional Autónoma de México, Cuernavaca, México; ^2^ Undergraduate Program in Genomic Sciences, Center for Genomic Sciences, Universidad Nacional Autónoma de México, Cuernavaca, México; ^3^ Department of Chemical Engineering, Universidad de Antioquia, Medellín, Colombia

**Keywords:** gene regulatory networks, organization, functional architecture, system principles, hierarchy, consistency, incompleteness, evolution

## Abstract

Synthetic biology aims to apply engineering principles for the rational, systematical design and construction of biological systems displaying functions that do not exist in nature or even building a cell from scratch. Understanding how molecular entities interconnect, work, and evolve in an organism is pivotal to this aim. Here, we summarize and discuss some historical organizing principles identified in bacterial gene regulatory networks. We propose a new layer, the concilion, which is the group of structural genes and their local regulators responsible for a single function that, organized hierarchically, coordinate a response in a way reminiscent of the deliberation and negotiation that take place in a council. We then highlight the importance that the network structure has, and discuss that the natural decomposition approach has unveiled the system-level elements shaping a common functional architecture governing bacterial regulatory networks. We discuss the incompleteness of gene regulatory networks and the need for network inference and benchmarking standardization. We point out the importance that using the network structural properties showed to improve network inference. We discuss the advances and controversies regarding the consistency between reconstructions of regulatory networks and expression data. We then discuss some perspectives on the necessity of studying regulatory networks, considering the interactions’ strength distribution, the challenges to studying these interactions’ strength, and the corresponding effects on network structure and dynamics. Finally, we explore the ability of evolutionary systems biology studies to provide insights into how evolution shapes functional architecture despite the high evolutionary plasticity of regulatory networks.

## Introduction

Synthetic biology aims to apply engineering principles for the rational, systematical design and construction of biological systems displaying functions that do not exist in nature or even building a cell from scratch (Abil and Danelon, 2020). To fulfill these ambitious goals, we not only need to understand how the various entities within a cell interact but also to identify the principles governing how the cellular systems interconnect, work, and evolve, as these are design cornerstones underpinning a successful rational design.

Whereas studying the whole set of molecular interactions across the different layers (e.g., transport, gene regulation, protein-protein interactions, metabolism, etc.) in a cell is necessary, it is not fully possible nowadays as current knowledge of the networks integrating the different layers is limited, and the integration of heterogeneous networks poses problems not yet solved. We thus focus on gene regulation as it is the key process that controls and integrates signals from all the other layers to cope with the environment.

Advances in understanding the inner workings of small regulatory circuits (i.e., network motifs) have provided good foundations to develop small synthetic circuits, but an understanding of the system principles governing the large-scale organization of complex biological networks is still elusive. However, these principles are pivotal to understanding how the organization of gene regulatory networks (GRNs) governs its possible dynamic outcomes (Ruklisa et al., 2019) and to enabling the successful integration of newly designed systems into the preexisting circuitry of molecular interactions in a chassis.

## The Basic Organizational Layer, Coupled Genes: The Operon

**FIGURE 1:**
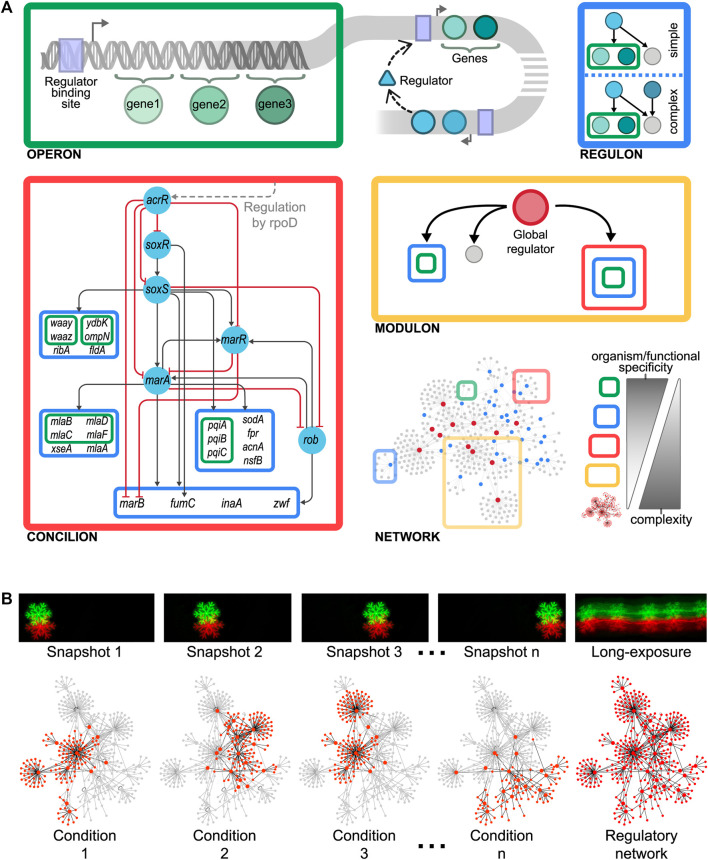
**(A)** Organizational layers shaping the modular hierarchy of the gene regulatory organization as gene < operon < regulon < concilion < modulon. A biological example of the here-proposed concilion is the “response to multiple stresses” module found in *E. coli* (Escorcia-Rodriguez et al., 2020). The grey dashed line shows that *acrR* is globally controlled by *rpoD*, which also controls other concilions and regulons (Figure 2A). The master regulators in this hierarchy are SoxR and SoxS, which respond to oxidative stress through sensing superoxide and nitric oxide. SoxS, MarA, and Rob bind as monomers to the same DNA site, a 20-bp degenerated sequence known as Mar/Sox/Rob box. The differential regulation of these genes could be archived by the degeneracy of their DNA binding sites or by the regulators’ concentration and the different affinities for the Mar/Sox/Rob box (Martin et al., 1999; Chubiz et al., 2012). The presence of several paralogous regulators (members of the AraC/XylS family) recognizing the same DNA binding site allows to archive a differential response by activating the same genes in response to different environmental cues (Martin et al., 2008). This phenomenon, known as commensurate regulon activation, enables bacteria to mount a proportionate response of the *marA*/*soxS*/*rob* regulon to the stress signal, keeping the number of activated genes to the minimum necessary to cope with prolonged stress (Martin et al., 2008; Wall et al., 2009). This balances the energetic cost of gene expression against the intensity of the stress. **(B)** Curated reconstructed regulatory networks merge many individual condition-specific subnetworks (such as picture snapshots) into a single network model thus capturing all the possible dynamic trajectories (such as a long-exposure photo does). Consequently, curated regulatory networks are not static representations of regulation, as they embed all the potential regulations that can occur thus constraining the large number of organizations a regulatory network could potentially have.

In 1960, Jacob *et al.* proposed the first genetic organizational level in the cell as a “unit of coordinated expression”, the operon (Jacob et al., 1960). This functional unit plays a key role in the hypothesis of the operator, explaining the polar effect occurring because of some mutations affecting the induction of enzymes needed to metabolize lactose in *Escherichia coli*. An operon comprises a set of adjacent genes that are regulated as a unit and co-transcribed into a single polycistronic mRNA (Jacob and Monod, 1961) (Figure 1A, top left). Genes composing an operon are usually functionally related (de Daruvar et al., 2002; Osbourn and Field, 2009) as they collaborate to attain a specific physiological function, although they commonly possess different biochemical activities. However, there are also cases of operons comprising genes without any apparent functional relation. In these cases, genes may be required in the same environmental conditions despite being involved in different pathways (Osbourn and Field, 2009), as if a special element, responsible for integrating, at the promoter level, disparate physiological responses, was possibly lurking there. While the operon solves the problem of co-regulating functionally related genes diminishing gene expression noise and ensuring more precise stoichiometry (Osbourn and Field, 2009), it has some limitations. First, some cellular processes involve too many genes. For example, anaerobic respiration in *E. coli* comprises more than 150 genes. An operon containing all these genes would encode a huge transcript whose transcription and processing, if possible, would be inefficient. Besides, these dozens of genetic products must be, not only expressed, but also precisely coordinated in time and quantity, something that an operon is unable to achieve.

## Coordinating Timing and Stoichiometry of Uncoupled Genes: The Regulon

A single regulatory protein may affect various promoters shaping what is defined as a regulon as was defined by Maas in 1964 (Maas, 1964). This organization enables the coordination of operons that are physically scattered throughout the genome. There are two types of regulons: simple and complex. Simple regulons are the set of genes, operons, or both regulated by a specific regulatory protein (Maas, 1964), whereas complex regulons are defined as the set of genes, operons, or both regulated by the same set of (two or more) regulatory proteins (Gutierrez-Rios et al., 2003) (Figure 1A, top right). As genes composing an operon are usually functionally related, the same holds for the operons controlled by a simple regulon. Besides, the expression of genes composing a regulon is not strictly coordinated, thus allowing variations in quantity and timing of synthesized products. These variations depend upon the concerted action of the respective promoters for each gene or operon in the regulon and the corresponding binding sites for their regulatory proteins. While regulons solve the organizational problems posed by operons, they open a new problem. How to control a single complex function that requires the coordinated expression of different regulons?

## The Power of Decentralized Global Coordination: The Modulon

**FIGURE 2:**
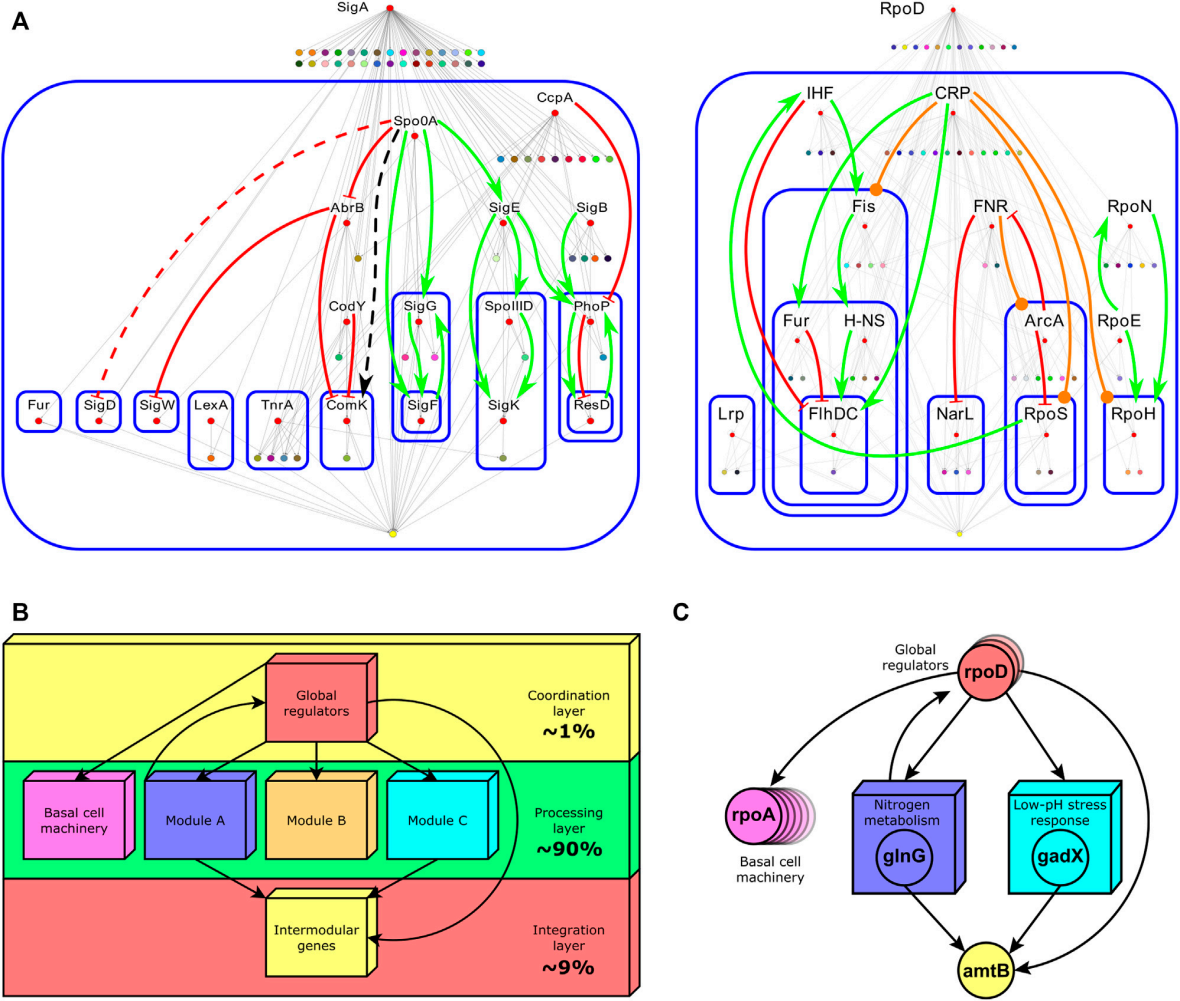
**(A)** Hierarchies identified by the theoretical pleiotropy approach for *B. subtilis* (left) and *E. coli* (right). Labeled red nodes are global regulators. Nodes composing modules were shrunk into a single colored node. At the bottom of each figure, the yellow node contains the set of intermodular genes. Continuous arrows (red for negative interactions, green for positive ones, orange for duals, and black for interactions whose sign is unknown) indicate regulatory interactions between global regulators. Blue rounded-corner rectangles bound hierarchical layers. For a detailed description of this figure, the reader is referred to the original caption (Freyre-Gonzalez et al., 2012)^1^. **(B)** The common functional architecture found across bacteria by the NDA. Percentages indicate the fraction of genes in the GRN composing that layer. **(C)** A biological example of each layer composing the functional architecture of the *E. coli* GRN. The global regulator *rpoD* is one of the several global regulators controlling genes in many modules (concilions and regulons). Global regulators also control many single genes or operons not regulated by local regulators (basal machinery). Two examples of modules, ‘Nitrogen metabolism’ and ‘Low-pH stress response’, are shown. They jointly control the intermodular gene *amtB via* the local regulators *glnG* (NtrC) and *gadX* (GadX). NtrC is the general regulator of the nitrogen assimilation pathway. GadX is one of the central regulators of the glutamate-dependent acid resistance system (GAD system). The *amtB* gene encodes an $NH_{4}^{+}$ antiporter. Disruption of this gene impaired the growth on ammonium only under acidic conditions. Ammonium is also a precursor of glutamate, which plays a central role in the GAD system. This shows that intermodular genes integrate disparate physiological responses coming from different modules.

The integration of single regulatory circuits into complex networks led Susan Gottesman to propose the existence of global regulatory proteins controlling these global networks in 1984 (Gottesman, 1984). In her seminal paper, she also provided a set of diagnostic criteria to identify this kind of regulator: 1) global regulators control a large number of genes, 2) the regulated genes are involved in more than one metabolic pathway, and 3) global regulators coordinate gene expression in response to a common need. Four years later, Iuchi and Lin defined the modulon as the set of operons, regulons, or both modulated—hence the word modulon, which has no relation to the term module—by a common pleiotropic regulatory protein (Iuchi and Lin, 1988) (Figure 1A, center right). Here, pleiotropy implies that operons and regulons under control are no longer functionally related. Therefore, mutations in the pleiotropic regulatory protein controlling the modulon give rise to alterations in multiple phenotypic traits in a cell, confirming that global regulators are involved in disparate physiological functions. A pleiotropic or global regulator is responsible for sensing and responding to signals of general interest for the cell such as DNA^1^ damage, stresses, or energy levels (Freyre-Gonzalez et al., 2008). Each global regulator shapes only one modulon and these could overlap by the co-regulation of some genes. Global regulators also shape a hierarchy comprising chains of command having each a specific physiology as has been previously reported for *E. coli* (Freyre-Gonzalez et al., 2008) and *Bacillus subtilis* (Freyre-Gonzalez et al., 2012) (Figure 2A). These chains of command modulate the local responses carried on, at the regulon level, by local regulators according to general interest environmental cues (e.g., low glucose, heat, high oxidizing power). Hence, modulons mostly shape a top-down hierarchy that could be seen as the global control device of the cell responsible for the coordination of lower functionally related structures. An interesting biological example of this global control device and its chains of command was outlined for the global regulator CtrA of Caulobacter crescentus (Laub et al., 2000).

## The Missing Piece: Coordinating a Single Function Using a Hierarchy of Local Regulators, the Concilion

Modularity is an organizing principle in the cell (Hartwell et al., 1999). Genomic islands (e.g., pathogenicity islands, secretion islands, antimicrobial resistance islands, and metabolic islands) and compartmentalization are clear examples of this. As we previously discussed, genes are grouped into operons, regulons, and modulons. All these are kinds of modules shaping the levels of the genetic organization. Indeed, regulons have been considered by far the ultimate level of genetic organization for functionally-related genes (Gutierrez-Rios et al., 2003). However, some complex processes, involving operons, regulons, or both devoted to closely related functions, require the coordinated expression, controlled in both time and quantity, of different regulons. Besides, processing genetic and environmental information may require both 1) dividing tasks into specialized processing units and 2) integrating the resulting information. For example, an antibiotics resistance module may comprise operons or regulons each responsible for providing resistance to different antibiotics. Hence, operons and regulons must be embedded into a complex structure that cannot be reduced into a simple regulon of regulons but that still is responsible for a unique, well-defined physiological function.

We defined this novel structure, previously only loosely named module, as the concilion [kon'si.li.on]. The term is derived from the Latin noun *concilium*, council or meeting, and the verb *conciliō*, to unite, to bring together. This refers to the group of structural genes and their local regulators responsible for a single function that, organized hierarchically, coordinates a response in a way reminiscent of the deliberation and negotiation that take place in a council (Figure 1A, bottom left). Concilions may be differentiated from regulons because the former exhibits interactions between their regulators resembling a hierarchical circuit that could even include some feedback and cross-regulation. Moreover, concilions do not contain any global regulator, they are local regulation devices devoted to a unique, well-defined function, contrary to modulons that include a global regulator by definition and control a diversity of functions. By analyzing a non-redundant set containing the most recent GRN for each of the 42 bacteria in Abasy Atlas, we found that, on average, roughly 17% of the modules identified by the natural decomposition approach (NDA, see next section) in a GRN are concilions. Furthermore, in the most recent reconstruction of the *E. coli* GRN (Abasy Atlas regnetid: 511145_v2020_sRDB18-13), we found that about 25% of the modules are concilions whereas the remainder modules are simple or complex regulons, highlighting the important role of the concilion in the functional architecture.

A biological example of this novel genetic organizational level is provided by the “response to multiple stresses” module found in *E. coli* (Escorcia-Rodriguez et al., 2020). This concilion comprises several regulons organized into a regulatory cascade mainly controlled by SoxR, SoxS, Rob, MarR, and MarA, which shapes a hierarchy regulating 22 structural genes, many of them regulated by two or more regulators, involved in the response of *E. coli* to stress from antibiotics, organic compounds, mechanical, oxidative, and xenobiotics (https://abasy.ccg.unam.mx/module?regnetid=511145_v2020_sRDB18-13_eStrong&class=39.2). Therefore, the different organizational layers shape the modular hierarchy of the gene regulatory organization as gene < operon < regulon < concilion < modulon. As we ascend in this hierarchy network complexity increases whereas functional and organism specificity decrease (Figure 1A, bottom right). This introduces at least two new problems for the study of genetic organization: 1) how a concilion can be identified, and 2) how the hierarchy governing these different genetic levels can be inferred.

## Unraveling the Common Functional Architecture and System Elements of Global GRNs

Studying the system dynamics of large-scale regulatory networks is challenging. Using a standard differential equations model to study the evolution in time of a system having thousands of interactions renders the model prohibitively complex. Moreover, despite the large availability of genomic data, incomplete knowledge of the system also hinders this goal (e.g., the poor availability of kinetic parameters). Therefore, as system complexity increases less detail must be included in the model (Bornholdt, 2005). On the other hand, the study of the system organization is fundamental as it constrains the possible dynamic outcomes (Ruklisa et al., 2019). Traditionally, one is interested in those genes responding to a particular condition, while this is interesting to study a specific response it is just an instantaneous snapshot of the system. The combinatorial nature of gene expression requires many individual condition-specific subnetworks (akin to picture snapshots) merged into a single network model to capture all the possible dynamic trajectories, in the way a long-exposure photo does (Figure 1B). This global network model is not a static representation of regulation, contrary to the specific-condition network. Instead, it embeds all the potential regulations that can occur, forming a regulatory landscape by constraining the large number of organizations a regulatory network could potentially have.

Curation efforts have allowed the reconstruction of updated regulatory networks for many organisms, alleviating the large-scale study of the architecture of regulatory networks. Further curation can help to improve the current reconstructions and even increase the number of organisms with an available reconstructed regulatory network. However, the massive curation of regulatory networks is limited by competitive funding with short grant cycles, which renders long-term funding, if available, uncertain, although alternative subscription-based funding models have been proposed (Reiser et al., 2016). Recently, Abasy Atlas (https://abasy.ccg.unam.mx) has gathered the largest collection of disambiguated and homogenized regulatory networks with experimentally validated interactions (Ibarra-Arellano et al., 2016). Such networks cover 42 bacteria distributed in nine species, including historical snapshots of the regulatory network reconstruction of some organisms at different stages of curation (Escorcia-Rodriguez et al., 2020). The construction of Abasy Atlas has exposed the poor knowledge we have about regulation in bacteria as only roughly 10% of the organisms in Abasy Atlas have a reconstructed regulatory network with interaction completeness > 65%. This statistic is based on a recent model developed to quantify the total number of interactions that the regulatory network of an organism will have according to its genome size (Campos and Freyre-Gonzalez, 2019; Escorcia-Rodriguez et al., 2020). This interaction completeness model is implemented and available in Abasy Atlas since version 2.2. Regulatory networks deposited in Abasy Atlas include different types of regulations (e.g., protein-DNA, small RNAs, and protein-protein interactions). Abasy Atlas also provides the system elements identified by the natural decomposition approach (NDA) that compose the functional architecture of a regulatory network.

The NDA leverages the global structure of a regulatory network to define mathematical diagnostic criteria and an algorithm to identify these system elements by the controlled decomposition of a network (Freyre-Gonzalez et al., 2008; Freyre-Gonzalez and Trevino-Quintanilla, 2010; Freyre-Gonzalez et al., 2012; Ibarra-Arellano et al., 2016; Freyre-Gonzalez and Tauch, 2017; Escorcia-Rodriguez et al., 2020; 2021). First, the *κ*-value is computed as the solution $\left(\sqrt[{\alpha +1}]{\alpha \gamma }\cdot k_{\mathrm{ou}t_{\max}}\right)$ to the equation $dC\left(k_{\mathrm{out}}\right)/dk_{\mathrm{out}}=-1$ , where $C\left(k_{\mathrm{out}}\right)=\gamma k_{\mathrm{out}}^{-\alpha }$ is the clustering coefficient distribution of a GRN as a function of the out-connectivity $\left(k_{\mathrm{out}}\right)$ and is obtained by robust least-squares fitting. The global regulators are identified as those having out-connectivity > *κ*-value. The global regulators and their interactions are removed from the network to naturally reveal the modules (remaining connected subgraphs) and the basal machinery (disconnected nodes). Intermodular genes are identified as structural genes (nodes having zero out-connectivity $\left(k_{\mathrm{out}}=0\right)$ and therefore no coding for regulators) being controlled by different modules and then integrating disparate physiological responses. For further details on the NDA methodology, please see Figures 1, 2 in both (Ibarra-Arellano et al., 2016; Freyre-Gonzalez and Tauch, 2017). Sensitivity analyses have shown that the global regulators are the most robust to network incompleteness, whereas the intermodular genes are the most labile. By focusing on the modular and basal machinery genes, it has been observed that the NDA is highly robust to incompleteness in the set of interactions and more labile to incompleteness in the set of genes. This suggests that NDA predictions from GRNs having high network genomic coverage are quite reliable (Freyre-Gonzalez and Tauch, 2017). These observations have been supported by analyzing historical snapshots of the *E. coli* GRN (Escorcia-Rodriguez et al., 2020). Additionally, an assessment of the NDA predictions obtained by using three network models of the *C. glutamicum* GRN with different confidence degrees, including the addition of small RNAs, and an analysis of historical snapshots, have also confirmed these observations (Escorcia-Rodriguez et al., 2021).

All together global regulators, modules comprising modular genes, basal machinery genes, and intermodular genes compose a non-pyramidal, three-tier, diamond-like hierarchy common to all the organisms in Abasy Atlas (Figure 2B). The diamond-like nature of the functional architecture follows from the asymmetry in the number of genes composing each layer. The coordination layer comprises roughly 1% of the genes in the network, whereas the processing layer, composed of modular and basal machinery genes, accounts for about 90%, and the integration layer comprises roughly 9%. The global regulators (coordination layer) modulate the expression of genes belonging to the two lower layers (processing and integration), whereas some feedback could occasionally occur between the processing and coordination layers. Modules identified by the NDA can be concilions or regulons, but neither modulons nor single operons. Nevertheless, modulons globally coordinate modules. Basal machinery genes account for the cell’s housekeeping functions and are controlled only by global regulators (Freyre-Gonzalez et al., 2012). Each module is responsible for a specific different function, whose combinatorial expression allows the cell to cope with a variety of environments. Remarkably, the NDA revealed that modules are locally independent meaning that there is no cross-regulation between them (Freyre-Gonzalez et al., 2012). Global regulators only coordinate the modules, and intermodular genes integrate some of their responses. Intermodular genes compose the integration layer. They were first identified by the NDA, they integrate, at the promoter level, the response of functionally disparate modules, and they thus enable the cell to cope with complex environments such as the assimilation of nitrogen under acidic conditions (Figure 2C) (Freyre-Gonzalez and Trevino-Quintanilla, 2010).

An alternative approach is to study expression data to elucidate the underlying network structures governing gene expression (Saelens et al., 2018). Recent works have applied independent component analysis (ICA) to transcription datasets to unravel the signals that govern gene expression in *E. coli*, *S. aureus*, and *B. subtilis* (Sastry et al., 2019; Rychel et al., 2021)*.* The analysis produces a series of so-called iModulons (unrelated to the traditional term modulon, see above), a group of genes that are governed by a certain signal. This signal in many cases can be assigned to a certain regulator, based on biological knowledge. A gene can be included in more than one iModulon and some iModulons are assigned to more than one regulator, which is consistent with the existence of complex regulons. This analysis partially reconstructs some of the known regulons of the network and even aids in predicting new regulatory interactions.

## Dealing With GRNs Incompleteness

From the perspective of rational synthetic biology, the top-down approach can be applied to identify disposable components in an organism using a global GRN (Lastiri-Pancardo et al., 2020). So far, not even the model organisms in gene regulation have a complete experimentally supported GRN (Escorcia-Rodriguez et al., 2020) because of the time and resource consumption needed for experimental validation and curation. Therefore, network inference is currently one of the best alternatives to reconstructing complete GRNs. However, it is a still-going challenge that, on one hand, has been approached through a plethora of transcriptomics-based strategies ranging from mechanistic models to machine learning, all of them with modest to poor results (Marbach et al., 2012). Network inference based on the identification of regulatory binding sites has performed significantly better (Zorro-Aranda et al., 2022), but it requires a prior network for its application. One way to deal with this limitation is to transfer regulatory information from one organism to another (Alkema et al., 2004). Nevertheless, this approach requires the organisms to be similar enough so the interactions are conserved (McCue et al., 2002; Escorcia-Rodriguez et al., 2021), and prior regulatory information for the source organism is still required. Inferences based on gene expression data have also benefited from the integration of biological information. For instance, the pre-selection of transcription factors (TFs) from experimental data constrains the number of potential inferences, and the application of structural properties of biological GRNs improves the assessment of the predictions (Zorro-Aranda et al., 2022). Other works have also shown improvements in the inference of regulatory networks integrating multiple omics data (Cheng et al., 2011; Banf and Rhee, 2017) and network structure (Castro et al., 2019). This suggests that the integration of biological data and network structure might be the approach to pursue in the inference of GRNs.

There is no straightforward nor standard way to infer a global regulatory network. A few precomputed inferences based on sequence or transcriptomics are scattered across the literature and organism-specific databases (Galan-Vasquez et al., 2020; Parise et al., 2020). Most of these inferences come from different approaches making it difficult to assess them. Besides, for the organisms with transcriptomic data, we need to gather and normalize the data to apply one of the top-ranking tools in previous assessments (Marbach et al., 2012). There exist databases hosting inferences of regulatory networks based on regulatory binding sites [e.g., PRECISE (Novichkov et al., 2013)]. However, these predictions have not been systematically assessed. We need to standardize the benchmarking of network inference tools with biological datasets and GRN gold standards used as reference. This way, we could keep pace with the rate of emerging methodologies. Moreover, the incompleteness of the GRN gold standards hinders proper assessment of inferred networks as actual true positive interactions are incorrectly labeled as false positive if they are not part of the current gold standard. We can leverage the constrained space for structural properties found in biological GRNs (Campos and Freyre-Gonzalez, 2019; Escorcia-Rodriguez et al., 2021) to verify if the inferred networks have similar properties.

Once we know the inferred networks behave as the biological ones, we can study their functional architecture and system-level components (Freyre-Gonzalez et al., 2012). Although the diamond-like structure has been found across all the organisms in Abasy Atlas, the system-level conservation has been quantitatively evaluated only between *E. coli* and *B. subtilis* (Freyre-Gonzalez et al., 2012), and *Corynebacterium glutamicum* and *Streptomyces coelicolor* (Zorro-Aranda et al., 2022). Future work assessing the conservation across all the available organisms and the robustness of the node classification to network incompleteness would shed light on the missing interactions for incomplete networks and their hierarchical role in the global network.

## Consistency of GRNs: Correlation Does Not Imply Causation

The consistency between GRNs and expression data has been previously studied by assuming a causal effect between the expression of the TFs and their target genes (TGs). Recent studies using expression data in *E. coli* and *C. glutamicum* have assessed this causal effect by using correlations to show a weak correlation of the known regulatory TF-TG pairs compared to all the possible random pairs as background (Larsen et al., 2019; Parise et al., 2021). Moreover, repressor interactions were associated with a positive correlation, rather than the expected negative correlation. This apparent inconsistency between GRNs and expression data may be explained by some molecular factors that cause known TF-TG pairs not to correlate well, e.g., the time delay between the stimulus and the regulatory response or TFs not being in their allosteric active configuration (Yu et al., 2003; Maier et al., 2009; Ghazalpour et al., 2011). Thus, we should not attempt to invalidate, through correlations of high-throughput expression data, reconstructed GRNs that are the result of experiments showing the physical binding of a TF to a DNA binding site. Further, expression data might capture false positive interactions and lead to an inherently noisy reconstruction of GRNs because found interactions are based on correlations and not necessarily causal.

Instead, an alternative approach, not yet reported, is to assess consistency within expression data considering the GRN architecture and organization. The functional architecture found in bacterial regulatory networks by the NDA (Escorcia-Rodriguez et al., 2020) (Figure 2B) proposes a robust partitioning of the network into physiologically correlated gene clusters and specific interaction roles for each regulatory interaction. This partitioning of the network may allow finding pairs of expressed genes that are significantly co-expressed across conditions by removing the noise in the previously unstructured set of interactions using a properly structured background. As mentioned above, expression data have been analyzed using ICA yielding significant biological results and partially reconstructing known regulons (Sastry et al., 2019; Rychel et al., 2021). This would be entirely impossible if expression data were completely inconsistent with the known structure of GRNs.

## Integrating Quantitative Information Into Network Representations of Gene Regulation

Weighted gene co-expression networks have been widely and successfully used to identify biologically relevant subgraphs, outperforming approaches based solely on network structure (Li et al., 2011; Niu et al., 2019; Farhadian et al., 2021). Perhaps including quantitative information in the network could aid structure-based approaches, such as the NDA, in discovering relevant modules. Optimally partitioning the network into subgraphs comprising strong interactions could also help identify sections of the network that can be modeled independently.

Research on GRNs has focused mainly on structural aspects, leaving out any quantitative information about how a certain regulator affects the expression of its targets. Although the modeling of gene expression dynamics based on Hill kinetics and differential equations becomes prohibitively complex as network size increases, simpler models could perhaps yield interesting information about how GRNs are globally organized. A first approach could be representing the network as a weighted graph, i.e., having each edge on the network assigned a certain weight that represents the strength of the interaction. The sole definition of what this strength would be (the affinity of the TF to its binding site, the TF-TG correlation, or some other measure) is itself a challenge as it is inherently related to the data used to quantify this information.

Integrating quantitative information into the network could yield valuable insights into the dynamical stability of the system as a whole or provide parameters with which to model small circuits within the network. Gene regulatory networks seem to be constrained in their density, tending towards lower values as network nodes increase, following a power law (Campos and Freyre-Gonzalez, 2019). In that study, the authors discuss that this restriction may stem from the necessity of dynamic stability, as predicted by the May-Wigner theorem (Gardner and Ashby, 1970; May, 1972). A 2018 study on the dynamics of phage *λ* demonstrated that, although some of its behavior can be solely explained by the structure of its network, the relative ordering of transcription factor binding site affinities determined modified behaviors of the attractors of the system (i.e., the set of the stable states the system arrives after perturbation) (Ruklisa et al., 2019). Advancing global network models from the purely qualitative to the quantitative are surely one of the ongoing challenges of biological network science, and essential to furthering our understanding of dynamic living systems.

## Evolution of GRNs From a System-Level Perspective

In a seminal paper in 1962, Herbert Simon proposed the idea that the evolution of a complex structure from simple elements must proceed through a hierarchy of potential stable subassemblies (Simon, 1962). In his parable of the two watchmakers, Simon argues that these hierarchical structures will evolve faster than non-hierarchical counterparts of similar size. Consequently, in the study of the evolution of complex structures such as complex biological networks, it is imperative to adopt an approach that considers how these potential stable subassemblies have played a role in their evolution. These subassemblies could be operons, regulons, concilions, or modulons in GRNs, all of them collectively referred to as systems hereafter. Therefore, for the study of the evolution of GRNs, we need a system-level approach.

Previous evolutionary studies have focused on the effect of gene duplication and horizontal gene transfer in the evolution of GRNs but without taking into account the network organization and how these mechanisms have given rise to its functional architecture (Madan Babu and Teichmann, 2003; Teichmann and Babu, 2004; Price et al., 2008). Further studies have assessed the conservation and evolution of GRNs by using networks inferred through orthology (Madan Babu et al., 2006) or biding sites prediction (Gonzalez Perez et al., 2008). All these advances have been properly summarized (Janga and Collado-Vides, 2007; Babu, 2010). Recently, some studies on eukaryotes have aimed to study how modularity evolves in developmental GRNs by using gene co-expression data (Peter and Davidson, 2011; Verd et al., 2019) or completely theoretical approaches (Espinosa-Soto and Wagner, 2010; Espinosa-Soto, 2018). An interesting study focuses on exploring the evolution of non-developmental GRNs (Defoort et al., 2018). By using genomic phylostratigraphy (Domazet-Loso et al., 2007), the authors explore the evolution at the level of small regulatory subgraphs (i.e., network motifs) of a mix of different types of GRNs in two eukaryotic organisms. Whereas this is an interesting study, the question of how evolution shapes the systems composing a GRN and its functional architecture is still an open question.

The lack of reliable methodologies to identify the system components integrating a GRN and the low completeness and standardization of GRNs have limited the study of its evolution from a systems perspective. Previous analyses have shown that the system elements proposed by the NDA are poorly conserved and that their evolution is possibly driven by evolutionary convergence (Freyre-Gonzalez et al., 2012). The recent availability of databases providing homogenized and standardized GRNs (Escorcia-Rodriguez et al., 2020), including the modules and system-level elements composing each GRN, provides the basis to explore how these systems have been shaped by evolution and whether stable subassemblies have arisen during the evolution of the currently known systems.

## Discussion

Without the study of the basic principles governing cell systems, it will be impossible for synthetic biology to become a true biological engineering discipline as has been defined by a European NEST (New and Emerging Science and Technology) High-Level Expert Group (European Commission, 2005; Pei et al., 2012) and repeatedly elsewhere (Serrano, 2007; Cheng and Lu, 2012; Bartley et al., 2017; Hanczyc, 2020). Even if the aim of being a true biological engineering discipline becomes elusive, the study of these fundamental principles is necessary to improve our basic understanding of biological complex systems (Schwille, 2011). All the themes presented in this paper are interconnected. Therefore, advance in one area affects the others. For example, having a model that describes the global organization of GRNs helps to delimit and guide their study in dynamics and evolution, as well as improve the understanding of their consistency with expression data. In turn, improvements in these subjects help to refine this model of the global network organization. Furthermore, all these topics together help to infer more and better GRNs incrementally improving our understanding of genomic regulation. Overall, during the last decade, some basic principles governing the still incomplete GRNs of a few organisms have been found. It is time to continue the research of these basic principles of biological complex networks to contribute to achieving rational synthetic biology.

## Data Availability

The original contributions presented in the study are included in https://abasy.ccg.unam.mx and the article/Supplementary Materials, further inquiries can be directed to the corresponding author.

## Footnotes

1 Reprinted from Journal of Biotechnology, 161:3, Julio A. Freyre-González, Luis G. Treviño-Quintanilla, Ilse A. Valtierra-Gutiérrez, Rosa María Gutiérrez-Ríos, José A. Alonso-Pavón, Prokaryotic regulatory systems biology: Common principles governing the functional architectures of *Bacillus subtilis* and *Escherichia coli* unveiled by the natural decomposition approach, 278-286, Copyright (2012), with permission from Elsevier.
