# Erratum: System principles governing the organization, architecture, dynamics, and evolution of gene regulatory networks

**DOI:** 10.3389/fbioe.2022.1091728

**Published:** 2022-12-02

**Authors:** Frontiers Production Office

**Affiliations:** Frontiers Media SA, Lausanne, Switzerland

**Keywords:** gene regulatory networks, organization, functional architecture, system principles, hierarchy, consistency, incompleteness, evolution

Due to a production error, a spelling error was introduced in an accession ID in the text. A correction has been made to the section **The Missing Piece: Coordinating a Single Function Using a Hierarchy of Local Regulators, the Concilion, paragraph 2.** The corrected paragraph appears below.

“We defined this novel structure, previously only loosely named module, as the concilion [kon’si.li.on]. The term is derived from the Latin noun *concilium*, council or meeting, and the verb *concili* ***ō***, to unite, to bring together. This refers to the group of structural genes and their local regulators responsible for a single function that, organized hierarchically, coordinates a response in a way reminiscent of the deliberation and negotiation that take place in a council (Figure 1A, bottom left). Concilions may be differentiated from regulons because the former exhibits interactions between their regulators resembling a hierarchical circuit that could even include some feedback and cross-regulation. Moreover, concilions do not contain any global regulator, they are local regulation devices devoted to a unique, well-defined function, contrary to modulons that include a global regulator by definition and control a diversity of functions. By analyzing a non-redundant set containing the most recent GRN for each of the 42 bacteria in Abasy Atlas, we found that, on average, roughly 17% of the modules identified by the natural decomposition approach (NDA, see next section) in a GRN are concilions. Furthermore, in the most recent reconstruction of the *E. coli* GRN (**Abasy Atlas regnetid: 511145_v2020_sRDB18-13**), we found that about 25% of the modules are concilions whereas the remainder modules are simple or complex regulons, highlighting the important role of the concilion in the functional architecture.”

The publisher apologizes for this mistake. The original version of this article has been updated.

